# The association between long working hours and work-related musculoskeletal symptoms of Korean wage workers: data from the fourth Korean working conditions survey (a cross-sectional study)

**DOI:** 10.1186/s40557-018-0278-0

**Published:** 2018-12-03

**Authors:** Jae-Gwang Lee, Guang Hwi Kim, Sung Won Jung, Sang Woo Kim, June-Hee Lee, Kyung-Jae Lee

**Affiliations:** Department of Occupational and Environmental Medicine, Soonchunhyang University Hospital, Seoul, South Korea

**Keywords:** Long working hours, Work-related musculoskeletal disorders (WMSDs), Korean working conditions survey (KWCS)

## Abstract

**Background:**

It has been reported that long working hours are hazardous to the workers’ health. Especially, work-related musculoskeletal disorders (WMSDs) have been considered as one of the significant health issues in workplace. The objective of this study was to identify the association between long working hours and work-related musculoskeletal symptoms.

**Methods:**

The analysis was conducted using data from the Fourth Korean Working Conditions Survey (KWCS). Subjects of this study were 24,783 wage workers and divided into three groups according to the weekly working hours, which were ≤ 40, 41–52 and > 52 h. The relationship between long working hours and work-related musculoskeletal symptoms was analyzed by multivariate logistic regression method after adjusting for general, occupational characteristics including specific working motions or postures and psychosocial factors.

**Results:**

Approximately 18.4% of subjects worked more than 52 h per week and 26.4 and 16.4% of male subjects and 33.0 and 23.4% of female subjects experienced work-related upper and lower limb pains, respectively, over the last 12 months. Moreover, the prevalence of upper and lower limb pain was increased in both genders as the weekly working hours increased. The odds ratios (ORs) of upper limb pain for those working 41–52 h and more than 52 h per week when adjusted for general, occupational characteristics including specific motions or postures and psychosocial factors were 1.36 and 1.40 for male workers and 1.26 and 1.66 for female workers compared to the reference group, respectively. Furthermore, ORs of lower limb pain for the same weekly working hour groups were 1.26 and 1.47 for male workers and 1.20 and 1.47 for female workers, respectively.

**Conclusions:**

Long working hours were significantly related to work-related musculoskeletal symptoms in Korean wage workers and appropriate interventions should be implemented to reduce long working hours that can negatively affect workers’ health.

## Background

As the industry develops increasingly, not only preexisting jobs have been expanded, but also new jobs have been come into being in plenty of fields of industry. Furthermore, for several jobs, there have been an extension of working hours and an introduction of night shift in duty to achieve an increase in productivity [1]. Especially in South Korea, not only changes in lifestyle of people made it possible to emerge stores which open 24 h such as convenience stores, cafés, or fast food stores but also the number of workers who work with long duty hours has increased because of comparatively higher wage of extended work or shift work [2]. According to the statistics from Organization for Economic Cooperation and Development (OECD), average weekly working hours of Korean workers in 2016 were 43.7 which was fourth highest and also exceeded by more than six hours compared to the average time of OECD countries [3]. Meanwhile, worker’s health issue which is caused by long working hours has received much attention because it is an important consideration to both employers and society in addition to workers themselves. Sickness of workers can cause decrease in efficiencies of work and subsequently reduce productivity in workplace as well as increase in socioeconomic burden [4]. Previous studies have shown the negative impact of long working hours on workers’ increased risk of hypertension [5, 6], coronary heart disease [7], stroke [8], anxiety [9], depression [10, 11], and occupational injuries [12, 13].

Meanwhile, prevalence of musculoskeletal disorders (MSDs) has been increased and considered as one of the significant health problems in workplace. According to the annual report about industrial injuries from Korean Ministry of Employment and Labor, work-related MSDs (WMSDs) accounted for 74.15, 71.80, and 68.41% of the occupational diseases in 2014, 2015, and 2016, respectively [14]. This implies that even though the proportion of the WMSDs among occupational diseases has tended to decrease slightly, they still occupy a significant portion in occupational diseases and need to be reduced much more.

Some studies have shown risk factors of WMSDs. Bernard et al. found epidemiological evidence of physical factors which are able to affect MSDs of upper extremity, lower extremity, and neck in their review article [15]. Several factors have been related to WMSDs such as awkward and/or sustained postures, excessive force, repetitive motion, and prolonged sitting or standing. Moreover, psychosocial factors such as occupational stress [16, 17], low social support, and job insecurity [18] are also considered to be related to WMSDs. Several studies have focused on the influence of work schedules on the prevalence of WMSDs. Two studies found that long work hours were associated with increased healthcare provider visits or short-term disability claims [19, 20]. Furthermore, Engkvist et al. [21] and Krause et al. [22] reported that long work hours were related to increase in back pain among nurses and transit operators, respectively. Another study showed that the combination of extended shifts and long working hours was linked to self-reported symptoms of the neck, shoulder, and back while controlling for age [23]. However, few studies have investigated the contribution of long working hours to WMSDs in South Korea. Shin at al. [24] showed that one of the risk factors of worker’s lower back pain was working more than 45 h per week and Lee [25] found in his cohort study that workers who consistently worked more than 48 h per week had a higher risk of lower back pain and the prevalence of lower back pain was decreased in case that working hours were reduced.

The purpose of this study was to identify the association between long working hours and work-related musculoskeletal symptoms of Korean wage workers using data from the Fourth Korean Working Conditions Survey (KWCS). While lower back pain is one of the typical musculoskeletal symptoms, we focused on the upper and lower limb pain as the work-related musculoskeletal symptoms because the upper and lower limb pains are important musculoskeletal symptoms and there have been few studies analyzing the relationship between long working hours and workers’ limb pain especially in Korean workers. In addition, the analysis was performed with gender stratification because the influence of a risk factor on specific type of occupation could differ in gender in industrial health researches [26, 27].

## Methods

### Study population

This study was based on data from the Fourth KWCS conducted by the Korea Occupational Safety and Health Agency in 2014. Subjects of KWCS were economically active Korean employed workers aged from 15 or more. Total 50,007 people responded the survey and 24,783 wage workers aged not fewer than 20 were selected for this study, excluding military personnel or workers employed in agriculture or forestry who occupy a small amount of respondents and those who refused to answer or left required fields of questionnaire blank. Because most workers aged below 20 work in part-time jobs temporarily and were low in number (*n* = 361), they were excluded from the study subjects [28, 29]. In addition, entering an aging society, there are jobs which have no legal retirement age such as security guards and therefore, aged workers are active in economic activity after their retirement. As a result, we did not set upper limit of age of the study population.

### Variables and measurements

#### General characteristics

Gender, age, educational status, and monthly income were considered as general characteristics of study population to analyze the influence on the work-related musculoskeletal symptoms. Age was divided into five groups of 20–29, 30–39, 40–49, 50–59 and more than 60. Educational status was categorized as middle school graduate or below, high school graduate, and college graduate or above. Also, monthly income was categorized as below 1,300,000, 1,300,000-1,999,000, 2,000,000-2,999,000, and 3,000,000 or more whose unit is Korean won.

#### Occupational characteristics

Occupational characteristics included type of occupation, employment status, shift work, scale of workplace, weekly working hours, and presence of specific working motions or postures. Type of occupation was divided into five groups of manager/professional, office worker, technician, service or sales worker, and manual worker. The manual workers included security guards, street cleaners, couriers, or parking guides. Also, employment status was categorized as regular workers and temporary/day labor workers. Shift work was simply divided into two groups, i.e., doing shift work or not. Scale of workplace was categorized based on the number of employees as below 50, 50–299, and 300 or more. Presence of specific motions or postures during work were evaluated using the following question: “Does your main paid job involve the followings?” and the specific motions or postures included lifting or moving people, carrying heavy loads, standing continuously, repetitive hand or arm movements, and working with computers. Study subjects were asked to check the corresponding proportion of time that each specific motion or posture occupies during work such as “all of the time”, “almost all of the time”, “around 3/4 of the time”, “around half of the time”, “around 1/4 of the time”, “almost never” or “never”. With the answers we dichotomized results into “No” if subject checked “never” or “Yes” if subject checked others.

Job stress and social support were considered as psychosocial characteristics. Each of them was asked as the following questions, respectively: “You experience stress in your work,” and “Your colleagues help and support you.” Subjects answered each question checking one of the examples such as “Always”, “Most of the time”, “Sometimes,” “Rarely”, or “Never” and were divided into low or high group according to the median score which was calculated by scoring each answers [30].

Weekly working hours, the independent variable of this study, were asked as the following question: “How many hours do you usually work per week in your main paid job?” Lunch break and commuting time were excluded from calculating the working hours. According to the Article 50 of the Korean Labor Standards Act, regular working hours per week in South Korea shall not exceed 40 h on average excluding recess hours; however, in case parties concerned reach agreement, the working hours per week may be extended up to 52 h [31]. Therefore, in this study, ‘long working hours’ were defined as more than 40 h per week and all study subjects were included in one of the following three groups in terms of working hours per week: less than or equal to 40 h, from 41 to 52 h, and more than 52 h.

#### Musculoskeletal symptoms

Musculoskeletal symptoms among study subjects, the dependent variable of this study, were investigated using the following question: “Over the last 12 months, did you have any of the following health problems?” Symptoms were largely divided into two groups. One of them was muscular pains in shoulders, neck and/or upper limbs (arms, elbow, wrists, hands etc.) and another was muscular pains in lower limbs (hips, legs, knees, feet etc.). In addition, we analyzed only results that subjects answered “Yes” in the following question: “Were the health problems related to your work?”

#### Statistical analysis

To determine factors contributing to weekly working hours and musculoskeletal symptoms in terms of the general and occupational characteristics of study subjects, the chi-square tests were performed. Moreover, multivariate logistic regression was implemented so as to analyze the relationship between weekly working hours and musculoskeletal symptoms by calculating odds ratios (ORs) and 95% confidential interval (CI) regarding two models: Model 1 was adjusted for gender, age, educational status, occupation, monthly income, employment status, shift work, and scale of workplace and Model 2 was adjusted for specific working motions or postures, job stress, and social support in addition to the covariates which were used in Model 1. All statistical analyses were performed using the SPSS version 18.0 (SPSS Inc., Chicago, IL, USA) and the level of statistical significance was set at *p* < 0.05.

## Results

### General and occupational characteristics of the study subjects

There were 11,890 (48.8%) female and 12,893 (52.0%) male subjects among total 24,783 study population and 53.5, 28.1 and 18.4% of all subjects worked ≤40, 41–52, and > 52 h per week, respectively (Table 1). The average age of the subjects was 43.4 years old and the age group of 30s (30.6%) and 60 years and more (23.9%) showed the largest proportions of working 41–52 and > 52 h per week, respectively. The greatest proportion of long working hours (> 40 h per week) was shown among workers whose final educational background was a high school (54.3%) and monthly income was in the range of 1,300,000-1,999,000 won (58.1%). In addition, 34.8% of technicians worked 41–52 h per week which was the largest proportion compared to the other occupations with respect to the same weekly working hours and 23.7% of service or sales workers and manual workers worked more than 52 h per week, which was the largest proportion regarding the same weekly working hours. Regular workers (48.7%), workers who had shift work (58.0%) and workers working in the workplace where the number of employees was under 50 (49.0%) showed the largest proportion of long working hours.

**Table 1:** General and occupational characteristics of subjects associated with the weekly working hours

Characteristics	Total(N,%)	Weekly working hours			*p*-value^*^
		≤40	41-52	> 52	
Total	24,783(100)	13,269(53.5)	6956(28.1)	4558(18.4)	
Gender					
Female	11,890(48.0)	6934(58.3)	3182(26.8)	1774(14.9)	< 0.001
Male	12,893(52.0)	6335(49.1)	3774(29.3)	2784(21.6)	
Age (years)					
20–29	3481(14.0)	1841(52.9)	990(28.4)	650(18.7)	< 0.001
30–39	6469(26.1)	3446(53.3)	1977(30.6)	1046(16.2)	
40–49	7280(29.4)	3959(54.4)	2103(28.9)	1218(16.7)	
50–59	4995(20.2)	2577(51.6)	1386(27.7)	1032(20.7)	
≥ 60	2558(10.3)	1446(56.5)	500(19.5)	612(23.9)	
Education					
Middle school graduate or below	2659(10.7)	1503(56.5)	552(20.8)	604(22.7)	< 0.001
High school graduate	9536(38.5)	4354(45.7)	2727(28.6)	2455(25.7)	
College graduate or above	12,588(50.8)	7412(58.9)	3677(29.2)	1499(11.9)	
Monthly income (KRW)					
< 1,300,000	5478(22.1)	3860(70.5)	984(18.0)	634(11.6)	< 0.001
1,300,000-1,999,000	6548(26.4)	2747(42.0)	2079(31.8)	1722(26.3)	
2,000,000-2,999,000	6964(28.1)	3308(47.5)	2282(32.8)	1374(19.7)	
≥ 3,000,000	5793(23.4)	3354(57.9)	1611(27.8)	828(14.3)	
Occupation					
Managers or Professionals	2594(10.5)	1731(66.7)	665(25.6)	198(7.6)	< 0.001
Office workers	6644(26.8)	4385(66.0)	1858(28.0)	401(6.0)	
Technicians	5438(21.9)	2279(41.9)	1890(34.8)	1269(23.3)	
Service or Sales workers	6525(26.3)	2877(44.1)	1808(27.7)	1840(23.7)	
Manual workers	3582(14.5)	1997(55.8)	735(20.5)	850(23.7)	
Employment status					
Regular	18,754(75.7)	9606(51.2)	5729(30.5)	3419(18.2)	< 0.001
Temporary or Day labor	6029(24.3)	3663(60.8)	1227(20.4)	1139(18.9)	
Shift work					
No	22,250(89.8)	12,206(54.9)	6242(28.1)	3802(17.1)	< 0.001
Yes	2533(10.2)	1063(42.0)	714(28.2)	756(29.8)	
Number of employees					
< 50	18,082(73.0)	9220(51.0)	5149(28.5)	3713(20.5)	< 0.001
50–299	4593(18.5)	2734(59.5)	1282(27.9)	577(12.6)	
≥ 300	2108(8.5)	1315(62.4)	525(24.9)	268(12.7)	

*calculated by chi-square test

In this study, 26.4 and 16.4% of male workers experienced work-related upper and lower limb pain over the last 12 months (Table 2) and 33.0 and 23.4% of female workers experienced the same symptoms, respectively, during the same period of time (Table 3). The proportions of having upper and lower limb pains in both genders tended to be increasing as the age of subjects was higher and the educational status or monthly income were lower except that the greatest proportion for upper limb pain of male workers was shown in the 1,300,000-1,999,000 won. In terms of occupation, the largest proportions were shown in the manual workers for upper and lower limb pains in both genders. The proportion of temporary or day labor workers with musculoskeletal symptoms was higher than that of the regular workers for both male and female workers. Furthermore, workers who did shift work and who worked in the workplaces where the number of employees was under 50 tended to experience upper and lower limb pain more compared to the workers who did not shift work and those working in the larger scale of workplaces for both genders. For the presence of specific working motions or postures, the proportions of having work-related musculoskeletal symptoms were shown to be larger when carrying heavy loads, standing continuously, and repetitive movement of arms or hands were included during work in both genders. Meanwhile, male and female workers who lift or carry people in their work process showed not much difference in prevalence of musculoskeletal symptoms compared to the workers who did not such working motions. As workers were under higher job stress and lower social support, they tended to have work-related musculoskeletal symptoms more.

**Table 2:** General and occupational characteristics of male subjects associated with the work-related musculoskeletal symptoms

Characteristics	Upper limb pain		*p*-value^*^	Lower limb pain		*p*-value^*^
	No(%)	Yes(%)		No(%)	Yes(%)	
Total	9493(73.6)	3400(26.4)		10,782(83.6)	2111(16.4)	
Age (years)						
20–29	1356(84.6)	246(15.4)	< 0.001	1461(91.2)	141(8.8)	< 0.001
30–39	2813(77.8)	804(22.2)		3199(88.4)	418(11.6)	
40–49	2623(73.2)	960(26.8)		3024(84.4)	559(15.6)	
50–59	1734(66.5)	872(33.5)		2008(77.1)	598(22.9)	
≥ 60	967(65.1)	518(34.9)		1090(73.4)	395(26.6)	
Education						
Middle school graduate or below	674(54.5)	563(45.5)	< 0.001	797(64.4)	440(35.6)	< 0.001
High school graduate	3153(67.9)	1489(32.1)		3684(79.4)	958(20.6)	
College graduate or above	5666(80.8)	1348(19.2)		6301(89.9)	713(10.2)	
Monthly income (KRW)						
< 1,300,000	1089(72.5)	413(27.5)	< 0.001	1170(77.9)	332(22.1)	< 0.001
1,300,000-1,999,000	1576(69.5)	692(30.5)		1790(78.9)	478(21.1)	
2,000,000-2,999,000	3183(71.6)	1261(28.4)		3707(83.4)	737(16.6)	
≥ 3,000,000	3645(77.9)	1034(22.1)		4115(87.9)	564(12.1)	
Occupation						
Managers or Professionals	1031(83.1)	209(16.9)	< 0.001	1142(92.1)	98(7.9)	< 0.001
Office workers	2981(84.7)	537(15.3)		3302(93.9)	216(6.1)	
Technicians	2731(64.8)	1486(35.2)		3255(77.2)	962(22.8)	
Service or Sales workers	1627(80.8)	386(19.2)		1752(87.0)	261(13.0)	
Manual workers	1123(59.0)	782(41.0)		1331(69.9)	574(30.1)	
Employment status						
Regular	7874(75.7)	2521(24.3)	< 0.001	8954(86.1)	1441(13.9)	< 0.001
Temporary or Day labor	1619(64.8)	879(35.2)		1828(73.2)	670(26.8)	
Shift work						
No	8340(74.3)	2890(25.7)	< 0.001	9475(84.4)	1755(15.6)	< 0.001
Yes	1153(69.3)	510(30.7)		1307(78.6)	356(21.4)	
Number of employees						
< 50	6134(72.1)	2375(27.9)	< 0.001	6976(82.0)	1533(18.0)	< 0.001
50–299	2082(75.3)	684(24.7)		2373(85.8)	393(14.2)	
≥ 300	1277(78.9)	341(21.1)		1433(88.6)	185(11.4)	
Working motion or posture						
Lifting or carrying people						
No	5605(73.8)	1990(26.2)	0.054	6364(83.8)	1231(16.2)	0.192
Yes	3888(73.4)	1410(26.6)		4418(83.4)	880(16.6)	
Carrying heavy loads						
No	3215(84.0)	614(16.0)	< 0.001	3503(91.5)	326(8.5)	< 0.001
Yes	6278(69.3)	2786(30.7)		7279(80.3)	1785(19.7)	
Standing continuously						
No	1935(84.1)	365(15.9)	< 0.001	2093(91.0)	207(9.0)	< 0.001
Yes	7558(71.3)	3035(28.7)		8689(82.0)	1904(18.0)	
Repetitive movement						
No	1789(88.4)	235(11.6)	< 0.001	1884(93.1)	140(6.9)	< 0.001
Yes	7704(70.9)	3165(29.1)		8898(81.9)	1971(18.1)	
Computer work						
No	2381(61.6)	1484(38.4)	< 0.001	2817(72.9)	1048(27.1)	< 0.001
Yes	7112(78.8)	1916(21.2)		7965(88.2)	1063(11.8)	
Job stress						
Low	2307(76.4)	713(23.6)	< 0.001	2550(84.4)	470(15.6)	< 0.001
High	7115(72.8)	2657(27.2)		8150(83.4)	1622(16.6)	
No response	71(70.3)	30(29.7)		82(81.2)	19(18.8)	
Social support						
High	8286(74.2)	2877(25.8)	< 0.001	9435(84.5)	1728(15.5)	< 0.001
Low	835(67.9)	395(32.1)		947(77.0)	283(23.0)	
No response	372(74.4)	128(25.6)		400(80.0)	100(20.0)	

*calculated by chi-square test

**Table 3:** General and occupational characteristics of female subjects associated with the work-related musculoskeletal symptoms

Characteristics	Upper limb pain		*p*-value^*^	Lower limb pain		*p*-value^*^
	No(%)	Yes(%)		No(%)	Yes(%)	
Total	7972(67.0)	3918(33.0)		9111(76.6)	2779(23.4)	
Age (years)						
20–29	1501(79.9)	378(20.1)	< 0.001	1638(87.2)	241(12.8)	< 0.001
30–39	2151(75.4)	701(24.6)		2428(85.1)	424(14.9)	
40–49	2484(67.2)	1213(32.8)		2856(77.3)	841(22.7)	
50–59	1320(55.3)	1069(44.7)		1603(67.1)	786(32.9)	
≥ 60	516(48.1)	557(51.9)		586(54.6)	487(45.4)	
Education						
Middle school graduate or below	636(44.7)	786(55.3)	< 0.001	750(52.7)	672(47.3)	< 0.001
High school graduate	3073(62.8)	1821(37.2)		3588(73.3)	1306(26.7)	
College graduate or above	4263(76.5)	1311(23.5)		4773(85.6)	801(14.4)	
Monthly income (KRW)						
< 1,300,000	2422(60.9)	1554(39.1)	< 0.001	2789(70.1)	1187(29.9)	< 0.001
1,300,000-1,999,000	2859(66.8)	1421(33.2)		3249(75.9)	1031(24.1)	
2,000,000-2,999,000	1856(73.7)	664(26.3)		2130(84.5)	390(15.5)	
≥ 3,000,000	835(75.0)	279(25.0)		943(84.6)	171(15.4)	
Occupation						
Managers or Professionals	1016(75.0)	338(25.0)	< 0.001	1123(82.9)	231(17.1)	< 0.001
Office workers	2471(79.0)	655(21.0)		2860(91.5)	266(8.5)	
Technicians	776(63.6)	445(36.4)		944(77.3)	277(22.7)	
Service or Sales workers	2908(64.5)	1604(35.5)		3194(70.8)	1318(29.2)	
Manual workers	801(47.8)	876(52.2)		990(59.0)	687(41.0)	
Employment status						
Regular	5743(68.7)	2616(31.3)	< 0.001	6624(79.2)	1735(20.8)	< 0.001
Temporary or Day labor	2229(63.1)	1302(36.9)		2487(70.4)	1044(29.6)	
Shift work						
No	7444(67.5)	3576(32.5)	< 0.001	8512(77.2)	2508(22.8)	< 0.001
Yes	528(60.7)	342(39.3)		599(68.9)	271(31.1)	
Number of employees						
< 50	6330(66.1)	3243(33.9)	< 0.001	7259(75.8)	2314(24.2)	< 0.001
50–299	1281(70.1)	546(29.9)		1452(79.5)	375(20.5)	
≥ 300	361(73.7)	129(26.3)		400(81.6)	90(18.4)	
Working motion or posture						
Lifting or carrying people						
No	4540(67.7)	2164(32.3)	0.054	5451(76.9)	1550(23.1)	0.192
Yes	3432(66.2)	1754(33.8)		3957(76.3)	1229(23.7)	
Carrying heavy loads						
No	3076(76.8)	929(23.2)	< 0.001	3440(85.9)	565(14.1)	< 0.001
Yes	4896(32.1)	2989(37.9)		819(79.1)	2155(20.9)	
Standing continuously						
No	1575(77.1)	468(22.9)	< 0.001	1809(88.5)	234(11.5)	< 0.001
Yes	6397(65.0)	3450(35.0)		7302(74.2)	2545(25.8)	
Repetitive movement						
No	1370(82.1)	299(17.9)	< 0.001	1467(87.9)	202(12.1)	< 0.001
Yes	6602(64.6)	3619(35.4)		7644(74.8)	2577(25.2)	
Computer work						
No	1962(53.5)	1706(46.5)	< 0.001	2333(63.6)	1335(36.4)	< 0.001
Yes	6010(73.1)	2212(26.9)		6778(84.4)	1444(17.6)	
Job stress						
Low	2130(69.7)	926(30.3)	0.001	2393(78.3)	663(21.7)	0.039
High	5773(66.2)	2950(33.8)		6634(76.1)	2089(23.9)	
No response	69(62.2)	42(37.8)		84(75.7)	27(24.3)	
Social support						
High	6650(67.6)	3191(32.4)	0.028	7643(77.7)	2198(22.3)	< 0.001
Low	842(64.5)	463(35.5)		931(71.3)	374(28.7)	
No response	480(64.5)	264(35.5)		537(72.2)	207(27.8)	

*calculated by chi-square test

### Working hours and work-related musculoskeletal symptoms

To investigate the relationship between the weekly working hours and work-related musculoskeletal symptoms, multivariate logistic regression analysis was implemented with gender stratification (Table 4). Compared with the reference group whose weekly working hours were ≤ 40, the ORs of prevalence of work-related upper limb pain for those working 41–52 h and > 52 h per week were 1.50 (95% CI 1.37–1.65) and 1.90 (95% CI 1.73–2.10), respectively, in male workers. On the other hand, the ORs of prevalence of upper limb pain in female workers were 1.22 (95% CI 1.12–1.33) and 1.96 (95% CI 1.76–2.18). With regard to lower limb pain, the ORs for those working 41–52 h and > 52 h per week were 1.39 (95% CI 1.24–1.55) and 2.09 (95% CI 1.87–2.34), respectively, in male workers. In female workers, the ORs of prevalence of lower limb pain were 1.17 (95% CI 1.06–1.29) and 1.98 (95% CI 1.77–2.22).

**Table 4:** Odds ratios and 95% confidence intervals of work-related musculoskeletal symptoms with gender stratification

	Weekly working hour	Upper limb pain				Lower limb pain			
		Male		Female		Male		Female	
		OR	95% CI	OR	95% CI	OR	95% CI	OR	95% CI
Crude	≤40	1	Reference	1	Reference	1	Reference	1	Reference
	41–52	1.50	1.37–1.65	1.22	1.16–1.33	1.39	1.24–1.55	1.17	1.06–1.29
	> 52	1.90	1.73–2.10	1.96	1.76–2.18	2.09	1.87–2.34	1.98	1.77–2.22
Model I^*^	≤40	1	Reference	1	Reference	1	Reference	1	Reference
	41–52	1.37	1.24–1.51	1.28	1.16–1.41	1.27	1.13–1.43	1.23	1.10–1.38
	> 52	1.47	1.32–1.64	1.77	1.57–2.00	1.52	1.34–1.73	1.56	1.40–1.72
Model II^**^	≤40	1	Reference	1	Reference	1	Reference	1	Reference
	41–52	1.36	1.23–1.50	1.26	1.14–1.39	1.26	1.11–1.42	1.20	1.07–1.35
	> 52	1.40	1.25–1.57	1.66	1.46–1.89	1.47	1.29–1.68	1.47	1.28–1.69

*Adjusted for gender, age, education, occupation, monthly income, employment status, shift work and number of employees

**Adjusted for gender, age, education, occupation, monthly income, employment status, shift work, number of employees, working motion or posture, job stress, and social support

When adjusted for general (gender, age, educational status, and monthly income) and occupational (occupation, employment status, shift work, and number of employees) characteristics, the ORs of upper limb pain were 1.37 (95% CI 1.24–1.51) and 1.47 (95% CI 1.32–1.64) for male workers working 41–52 h and > 52 h per week, respectively in Model 1. Furthermore, the ORs of male workers for the same weekly working hour groups were 1.27 (95% CI 1.13–1.43) and 1.52 (95% CI 1.34–1.73), respectively, regarding lower limb pain. On the other hand, the ORs of female workers were 1.28 (95% CI 1.16–1.41) and 1.77 (95% CI 1.57–2.00) for upper limb pain and 1.23 (95% CI 1.10–1.38) and 1.60 (95% CI 1.40–1.82) for lower limb pain in Model 1.

In addition to the characteristics which were adjusted in Model 1, specific working motions or postures (lifting or carrying people, carrying heavy loads, standing continuously, repetitive movement of arm or hands and computer work) and psychosocial factors (job stress and social support) were also adjusted in Model 2. The ORs of upper limb pain were 1.36 (95% CI 1.23–1.50) and 1.40 (95% CI 1.25–1.57) for male workers working 41–52 h and > 52 h per week, respectively. Also, the ORs of lower limb pain in male workers were 1.26 (95% CI 1.11–1.42) and 1.47 (95% CI 1.29–1.68) for those working 41–52 h and > 52 h per week, respectively. On the other hand, the ORs of upper limb pain were 1.26 (95% CI 1.14–1.39) and 1.66 (95% CI 1.46–1.89) for female workers working 41–52 h and > 52 h per week and the ORs of lower limb pain were 1.20 (95% CI 1.07–1.35) and 1.47 (95% CI 1.28–1.69) for the same weekly working hour groups in female workers, respectively.

### Age groups and occupation of study subjects

**Fig. 1:**
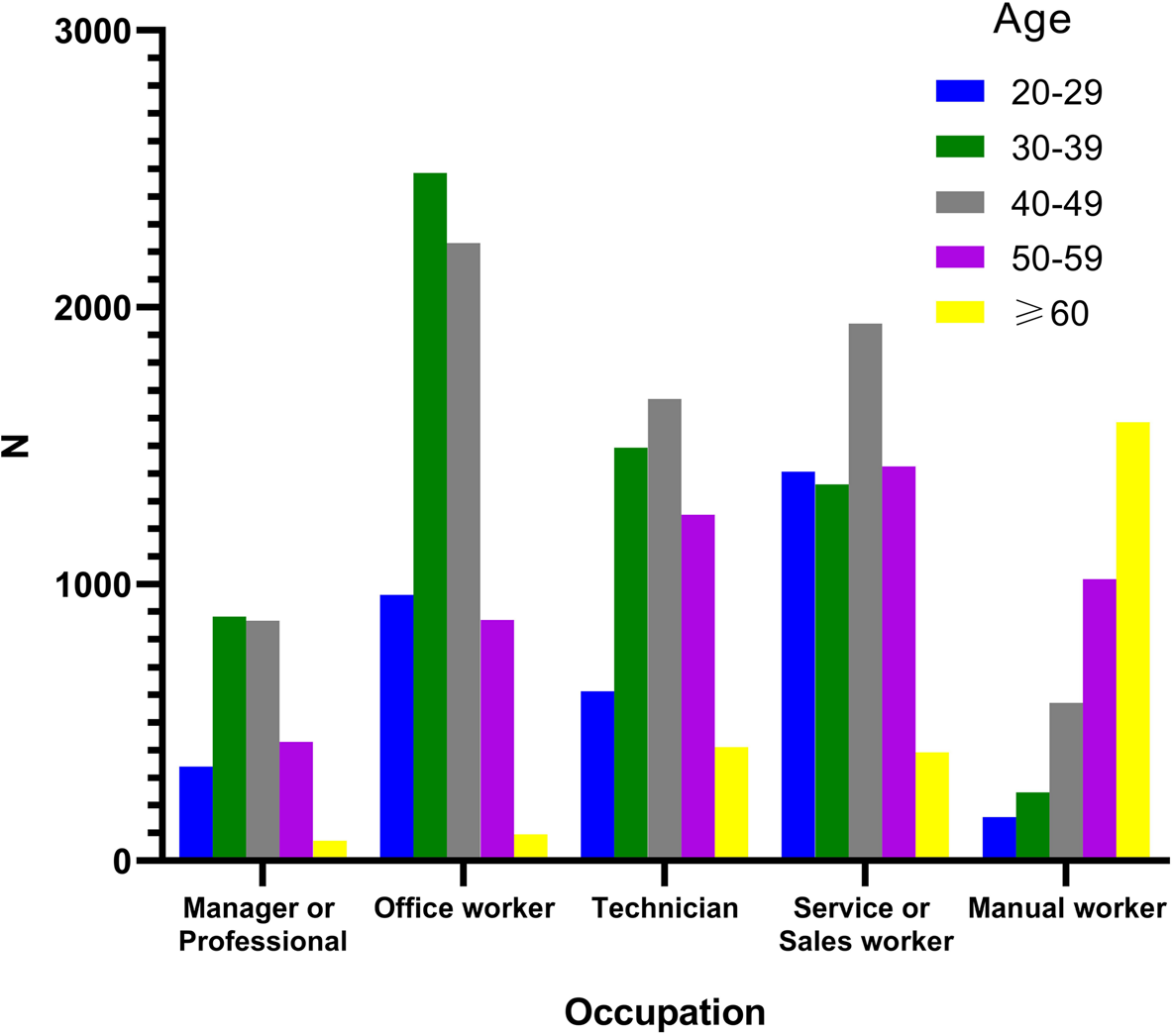
Relationship between age groups and occupations of subjects. Blue bar indicates the age group of 20–29 years. Green bar indicates the age group of 30–39 years. Grey bar indicates the age group of 40–49 years. Purple bar indicates the age group of 50–59 years. Yellow bar indicates the age group of 60 years and more

To investigate the distribution of age according to the occupation of study subjects, frequency analysis was performed (Fig. 1). Age group of 30–39 (green bar) and 40–49 (grey bar) occupied the greater proportions in managers or professionals, office workers, and technicians than any other occupations. Among service or sales workers, the age group of 40–49 showed the largest proportion (29.7%) and the age group of 60 years and more (yellow bar) was the most prevalent (44.3%) among manual workers. On the other hand, among age group of 60 years and more, manual workers (62.0%) showed the largest proportion than any other occupations.

## Discussion

In this study, we investigated the association between long working hours and work-related musculoskeletal symptoms among Korean wage workers. The results of analysis showed that as the working hours per week increased, the prevalence of upper and lower limb pain that workers experienced were also higher compared to the reference group of weekly working hours. The result also remained valid when adjusted for general and occupational characteristics in Model 1 and 2. Therefore, we found that long working hours independently increased workers’ prevalence of work-related musculoskeletal symptoms. This finding is also consistent with previous studies showing the relationship between the long working hours and WMSDs. Data from 24 years of follow-up has shown that the overtime work was associated with the diagnosis of shoulder disorders in women workers (Prevalence ratio [PR] 2.7; 95% CI 1.1–6.9) [32]. Furthermore, it was reported that working more than 13 h per day was one of the risk factors significantly related to neck, shoulder and back disorders in nurses (OR 1.94, OR 1.87, and OR 1.87 for neck, shoulder and back, respectively) [33]. Working 48 h and more per week was also shown to be associated with the elevated risk of back pain of those working in small and medium-sized 26 manufacturing companies (OR 1.98; 95% CI 1.02–3.83) [25].

WMSDs are known to be in strong association with the physical demands such as repetitive movement, awkward postures, and heavy lifting or pushing in the job [15]. The relationship between long working hours and the risk of WMSDs can be explained by the hypothesis that as the working hours increase, time exposed to the physical demands during work increases as well and this consequently could affect the higher prevalence of musculoskeletal diseases. In addition to such an ergonomic aspect, increase in working hours can cause relative reduction in recovery time of accumulated fatigue and leisure time to relieve stresses [2]. As a result, such factors complexly and cumulatively influence on worker’s musculoskeletal system and finally could induce WMSDs.

Another important finding of this study was that the proportion of workers working more than 52 h per week among the age group of 60 years and more (23.9%) was larger than that of other age groups. Furthermore, the type of occupation occupying the largest proportion among the age group of 60 years and more was the manual work (62.0%) when analyzing the distribution of occupations with regard to each age group. Considering the result that prevalence of musculoskeletal symptoms was the highest in the age group of 60 years and more and in the manual workers, these results imply that aged workers are more vulnerable to WMSDs because physical demands which can be a high burden to worker’s body are relatively higher in manual workers than any other occupations and the old age itself even increases the risk of WMSDs in that aged workers generally have worked for longer period of times than younger workers, so there could be the cumulative effect. Therefore, it is important to draw up any preventive measures or intervention programs to decrease WMSDs especially for aged workers. Moreover, the social structure in which aged people have a lot of physical labor should be changed.

Comparing the prevalence of work-related musculoskeletal symptoms of male workers with that of female workers, proportion of having work-related upper limb pain was larger in female workers than male workers and also larger in female workers for lower limb pain. This result is consistent with previous studies showing that the prevalence of work-related musculoskeletal symptoms was more frequent in female workers [34, 35]. Factors that increase the prevalence of musculoskeletal symptoms in female workers could be the burden of housework which women mostly take charge of other than men, tendency to express symptoms exaggeratingly in women and physiologic features that make women more vulnerable to musculoskeletal disease such as strength of muscles, difference of muscle fiber type and distribution, difference in hormones, and pregnancy [36]. On the other hand, except that the ORs of upper limb pain for female workers were shown to be higher than those for male workers as the weekly working hour exceeded 52 h, we found that the ORs of musculoskeletal symptoms for female workers were not always higher than those for male workers as the weekly working hour increased.

There are a few limitations in this study. First, while we showed the association between long working hours and work-related musculoskeletal symptoms, the results do not explain the causal relationship between them because this study was designed as a cross-sectional study. To identify the causality or temporal relationship between long working hours and musculoskeletal symptoms, further longitudinal studies should be performed. Second, this study was based on the Fourth KWCS which consists of a self-report questionnaire and therefore, there was a possibility of an information bias. Third, there might be other personal factors such as height, weight, exercise, or previous history of musculoskeletal diseases, which could affect the prevalence of musculoskeletal symptoms. However, such factors were not considered all in this study because of data limitations. Fourth, musculoskeletal symptoms analyzed in this study do not exactly mean the musculoskeletal disease because ‘symptoms’ are based on the subjective feelings of individuals, but ‘diseases’ are based on the objective diagnostic criteria. However, it is meaningful to analyze the prevalence of musculoskeletal symptoms in workplaces to prevent the occurrence of WMSDs because almost all musculoskeletal symptoms are accompanied by or come before the musculoskeletal diseases.

Despite these limitations, there are several strengths in this study. First, the data, KWCS, which we used is a representative national survey that investigated working conditions and worker’s health issue and provides reliable samples of Korean workers. Second, different from previous studies which limited in the specific occupational group, this study showed the relationship between long working hours and work-related musculoskeletal symptoms for various types of occupation. Third, there have been few studies about the association between long working hours and work-related upper and lower limb symptoms in Korea, thus this study can be a valuable reference for future researches.

## Conclusions

In conclusion, long working hours were associated with musculoskeletal symptoms in Korean wage workers. Further studies are necessary to find the concrete mechanism by which long working hours affect the prevalence of WMSDs and to show the causal relationship between them. Moreover, appropriate interventions should be implemented to reduce long working hours that can affect workers’ health and the optimal reference working hours should be set because the legal working hours differ from country to country.

## Abbreviations

CI: Confidence Interval
KRW: Korean Won
KWCS: Korean Working Conditions Survey
OECD: Organization for Economic Cooperation and Development
OR: Odds Ratio
PR: Prevalence Ratio
WMSD: Work-related Musculoskeletal Disorder

## References

[1] Costa Giovanni, Haus Erhard, Stevens Richard (2010). Shift work and cancer – considerations on rationale, mechanisms, and epidemiology. Scandinavian Journal of Work, Environment & Health.

[2] Caruso CC, Bushnell T, Eggerth D, Heitmann A, Kojola B, Newman K, Rosa RR, Sauter SL, Vila B (2006). Long working hours, safety, and health: toward a National Research Agenda. Am J Ind Med.

[3] Average usual weekly hours worked on the main job. OECD Statistics Available: [https://stats.oecd.org/Index.aspx?DataSetCode=AVE_HRS]. Accessed 3 Nov 2017.

[4] Caruso CC (2006). Possible broad impacts of long work hours. Ind Health.

[5] Nakanishi N, Yoshida H, Nagano K, Kawashimo H, Nakamura K, Tatara K (2001). Long working hours and risk for hypertension in Japanese male white collar workers. J Epidemiol Community Health.

[6] Yang H, Schnall PL, Jauregui M, Su T-C, Baker D (2006). Work hours and self-reported hypertension among working people in California. Hypertension.

[7] Virtanen M, Heikkilä K, Jokela M, Ferrie JE, Batty GD, Vahtera J, Kivimäki M (2012). Long working hours and coronary heart disease: a systematic review and meta-analysis. Am J Epidemiol.

[8] Kivimäki M, Jokela M, Nyberg ST, Singh-Manoux A, Fransson EI, Alfredsson L, Bjorner JB, Borritz M, Burr H, Casini A (2015). Long working hours and risk of coronary heart disease and stroke: a systematic review and meta-analysis of published and unpublished data for 603 838 individuals. Lancet.

[9] Kleppa E, Sanne B, Tell GS (2008). Working overtime is associated with anxiety and depression: the Hordaland health study. J Occup Environ Med.

[10] Amagasa T, Nakayama T (2013). Relationship between long working hours and depression: a 3-year longitudinal study of clerical workers. J Occup Environ Med.

[11] Virtanen M, Ferrie JE, Singh-Manoux A, Shipley MJ, Stansfeld SA, Marmot MG, Ahola K, Vahtera J, Kivimäki M (2011). Long working hours and symptoms of anxiety and depression: a 5-year follow-up of the Whitehall II study. Psychol Med.

[12] Dembe AE, Erickson JB, Delbos RG, Banks SM (2005). The impact of overtime and long work hours on occupational injuries and illnesses: new evidence from the United States. Occup Environ Med.

[13] Folkard S, Lombardi DA (2006). Modeling the impact of the components of long work hours on injuries and “accidents”. Am J Ind Med.

[14] Ministry of Employment and Labor. Yearbooks of industrial accident. Available**:** [http://www.kosha.or.kr/board.do?menuId=554]. Accessed 10 Nov 2017.

[15] Bernard BP, Putz-Anderson V (1997). Musculoskeletal disorders and workplace factors; a critical review of epidemiologic evidence for work-related musculoskeletal disorders of the neck, upper extremity, and low back.

[16] Kim MG, Kim K-S, Ryoo J-H, Yoo S-W (2013). Relationship between occupational stress and work-related musculoskeletal disorders in Korean male firefighters. Annals of occupational and environmental medicine.

[17] Leino P (1989). Symptoms of stress predict musculoskeletal disorders. J Epidemiol Community Health.

[18] Amin NA, Nordin R, Fatt QK, Noah RM, Oxley J (2014). Relationship between psychosocial risk factors and work-related musculoskeletal disorders among public hospital nurses in Malaysia. Annals of occupational and environmental medicine.

[19] Josephson M, Ahlberg G, Härenstam A, Svensson H, Theorell T, Wiktorin C, Vingård E (2003). Paid and unpaid work, and its relation to low back and neck/shoulder disorders among women. Women & health.

[20] O'Brien-Pallas L, Shamian J, Thomson D, Alksnis C, Koehoorn M, Kerr M, Bruce S (2004). Work-related disability in Canadian nurses. J Nurs Scholarsh.

[21] Engkvist I-L, Hjelm EW, Hagberg M, Menckel E, Ekenvall L (2000). Risk indicators for reported overexertion back injuries among female nursing personnel. Epidemiology.

[22] Krause N, Rugulies R, Ragland DR, Syme SL (2004). Physical workload, ergonomic problems, and incidence of low back injury: a 7.5-year prospective study of San Francisco transit operators. Am J Ind Med.

[23] Lipscomb Jane A, Trinkoff Alison M, Geiger-Brown Jeanne, Brady Barbara (2002). Work-schedule characteristics and reported musculoskeletal disorders of registered nurses. Scandinavian Journal of Work, Environment & Health.

[24] Shin KS, Chung YK, Lee HE (2012). Prevalence and risk factors of work-related low back pain among operators and drivers of transportation vehicle. Korean Journal of Occupational and Environmental Medicine.

[25] Lee D. The impact of long working hours and shift work on incidence of low Back pain : 3 years follow-up survey. Seoul National University, Department of Industrial Health. 2013.

[26] Messing K, Punnett L, Bond M, Alexanderson K, Pyle J, Zahm S, Wegman D, Stock SR, de Grosbois S (2003). Be the fairest of them all: challenges and recommendations for the treatment of gender in occupational health research. Am J Ind Med.

[27] Yoon J, Sung H, Kim Y, Kim S-S (2015). The relationship between experience of workplace violence and musculoskeletal pain among wage Workers in South Korea. Journal of Korean Society of Occupational and Environmental Hygiene.

[28] Jung SW, Lee K-J, Lee HS, Kim GH, Lee JG, Kim JJ, Lee J-H (2017). Relationship of activities outside work to sleep and depression/anxiety disorders in Korean workers: the 4th Korean working condition survey. Annals of occupational and environmental medicine.

[29] Seok H, Yoon J-H, Lee W, Lee J-H, Jung PK, Kim I, Won J-U, Roh J (2014). The association between concealing emotions at work and medical utilization in Korea. Annals of occupational and environmental medicine.

[30] Yoo T, Ye B, Kim J-I, Park S (2016). Relationship of workplace violence and perpetrators on sleep disturbance-data from the 4th Korean working conditions survey. Annals of occupational and environmental medicine.

[31] Ministry of Employment and Labor: Chapter IV: Working Hours and Recess. In Labor Standard Act. Available: [http://www.moel.go.kr/english/download_eng.jsp?type=&file=(31)LABORSTANDARDSACT_2012.pdf]. Accessed 12 Nov 2017.

[32] Fredriksson K, Alfredsson L, Köster M, Thorbjörnsson CB, Toomingas A, Torgén M, Kilbom A (1999). Risk factors for neck and upper limb disorders: results from 24 years of follow up. Occup Environ Med.

[33] Trinkoff AM, Le R, Geiger-Brown J, Lipscomb J, Lang G (2006). Longitudinal relationship of work hours, mandatory overtime, and on-call to musculoskeletal problems in nurses. Am J Ind Med.

[34] Kim BK, Park CY, Yim HW, Koo JW, Lee KS (2005). Selection of a high risk group and the effectiveness of an exercise program on musculoskeletal symptoms in small and medium sized enterprises. Korean Journal of Occupational and Environmental Medicine.

[35] Park SG, Lee JY (2004). Characteristics and odds ratio of work related musculoskeletal disorders according to job classification in small-to-medium-sized enterprises. Korean Journal of Occupational and Environmental Medicine.

[36] Kim HR, Won JU, Song JS, Kim CN, Kim HS, Roh JH (2003). Pain related factors in upper extremities among hospital workers using video display terminals. Korean Journal of Occupational and Environmental Medicine.

